# Inhibition of overexpression of Giα proteins and nitroxidative stress contribute to sodium nitroprusside‐induced attenuation of high blood pressure in SHR

**DOI:** 10.14814/phy2.13658

**Published:** 2018-03-29

**Authors:** Ekhtear Hossain, Oli Sarkar, Yuan Li, Madhu B. Anand‐Srivastava

**Affiliations:** ^1^ Department of Pharmacology and Physiology Faculty of Medicine University of Montreal Montreal Canada

**Keywords:** cGMP, Giα proteins, NO, oxidative stress, SHR

## Abstract

We earlier showed that vascular smooth muscle cells (VSMC) from spontaneously hypertensive rats (SHR) exhibit enhanced expression of Giα proteins which was attributed to the decreased levels of nitric oxide (NO), because elevation of the intracellular levels of NO by NO donors; sodium nitroprusside (SNP) and S‐Nitroso‐N‐acetyl‐DL‐penicillamine (SNAP), attenuated the enhanced expression of Giα proteins. Since the enhanced expression of Giα proteins is implicated in the pathogenesis of hypertension, the present study was undertaken to investigate if treatment of SHR with SNP could also attenuate the development of high blood pressure (BP) and explore the underlying molecular mechanisms. Intraperitoneal injection of SNP at a concentration of 0.5 mg/kg body weight twice a week for 2 weeks into SHR attenuated the high blood pressure by about 80 mmHg without affecting the BP in WKY rats. SNP treatment also attenuated the enhanced levels of superoxide anion (O_2_^−^), hydrogen peroxide (H_2_O_2_), peroxynitrite (ONOO ^−^), and NADPH oxidase activity in VSMC from SHR to control levels. In addition, the overexpression of different subunits of NADPH oxidase; Nox‐1, Nox‐2, Nox‐4, P^22phox^, and P^47phox^, and Giα proteins in VSMC from SHR were also attenuated by SNP treatment. On the other hand, SNP treatment augmented the decreased levels of intracellular NO, eNOS, and cGMP in VSMC from SHR. These results suggest that SNP treatment attenuates the development of high BP in SHR through the elevation of intracellular levels of cGMP and inhibition of the enhanced levels of Giα proteins and nitroxidative stress.

## Introduction

Guanine nucleotide‐binding regulatory proteins (G proteins) play an important role in the regulation of a variety of physiological functions including blood pressure through the activation of various signal transduction systems, including the adenylyl cyclase/cAMP system (Rodbell et al. 1971). The activity of adenylyl cyclase is regulated by stimulatory (Gs) and inhibitory (Gi) G proteins (Fleming et al. 1992; Gilman 1984; Neer 1995). G proteins are heterotrimeric and composed of *α*,* β*, and *γ* subunits. Molecular cloning has revealed four different isoforms of Gsα resulting from differential splicing of one gene (Bray et al. 1986; Robishaw et al. 1986) and three distinct isoforms of Giα: Giα‐1, Giα‐2, and Giα‐3 encoded by three different genes (Itoh et al. 1988).

The Gi protein and associated adenylyl cyclase signaling has been shown to be implicated in a variety of cellular functions including vascular tone (Yatani et al. 1988), cell proliferation (Bou Daou et al. 2016) etc., which play an important role in the regulation of blood pressure. Earlier studies have shown the overexpression of Giα proteins in cardiovascular tissues from genetic spontaneously hypertensive rats (SHR) as well as from other models of experimentally induced hypertensive rats (HR), including deoxycorticosterone acetate (DOCA)‐Salt, one‐kidney, one‐clip (1K‐1C) and N (omega)‐nitro‐L‐arginine methyl ester (L‐NAME) HR (Anand‐Srivastava 1992; Anand‐Srivastava et al. 1993; Bohm et al. 1992; Di Fusco and Anand‐Srivastava 2000; Ge et al. 2006; Thibault and Anand‐Srivastava 1992). The increased expression of Gi proteins was shown to be the contributing factor in the pathogenesis of hypertension in SHR and DOCA‐Salt HR (Marcil et al. 1998, 1997). In addition, the studies showing that inactivation of Giα proteins in prehypertensive rats (2 weeks old SHR) by single injection of pertussis toxin (PT) (Li and Anand‐Srivastava 2002) or knocking down of Giα‐2 protein by antisense treatment (Ali El‐Basyuni et al. 2016) prevented the development of high blood pressure further supported the role of enhanced expression of Gi proteins in the pathogenesis of hypertension.

Enhanced oxidative stress has been shown to play a critical role in the pathogenesis of cardiovascular disease including hypertension (Gomez Sandoval and Anand‐Srivastava 2011; Lappas et al. 2005). We earlier showed that vascular smooth muscle cells (VSMC) from SHR exhibit enhanced oxidative stress due to the overproduction of superoxide anion (O_2_^−^), increased activity of NADPH oxidase, and enhanced expression of NADPH oxidase subunits (Li et al. 2014; Saha et al. 2008; Sarkar et al. 2017) that contributes to the enhanced expression of Giα proteins in SHR (Lappas et al. 2005).

Nitric oxide (NO), a diffusible messenger has been shown to play a role in the regulation of a variety of physiological functions, including vasorelaxation and blood pressure (Lane and Gross 1999; Tonelli et al. 2013). We and others have shown a decreased production/bioavailability of NO associated with hypertension (Chou et al. 1998; Crabos et al. 1997; Elks et al. 2011; Li et al. 2014), which may be attributed to the decreased expression of endothelial nitric oxide synthase (eNOS) and to the increased levels of O_2_^−^ leading to the maintenance of the elevated peripheral resistance and thereby elevated BP (Pacher et al. 2007). We also showed earlier that treatment of rats with NO synthase inhibitor L‐NAME resulted in the enhanced expression of Giα proteins and increased blood pressure (Di Fusco and Anand‐Srivastava 2000; Hashim and Anand‐Srivastava 2004). These results suggest that decreased levels of NO in L‐NAME‐induced hypertensive rats may be responsible for the enhanced expression of Giα proteins and thereby hypertension. Sodium nitroprusside (SNP) and SNAP; NO donors were shown to decrease the expression of Giα proteins in VSMC (Arejian et al. 2009; Bassil and Anand‐Srivastava 2006) as well as the overexpression of Giα proteins in aortic VSMC from SHR (Sarkar et al. 2017). Taken together, it may be possible that in vivo treatment of SHR with NO donors attenuates the high blood pressure due to their ability to decrease the enhanced expression of Giα proteins which has been shown as the contributing factor in the pathogenesis of hypertension. The present study was therefore undertaken to investigate the effect of in vivo treatment of SHR with NO donor; SNP on the development of high blood pressure (BP) and to explore the underlying molecular mechanisms for this effect.

## Materials and Methods

### Chemicals

Sodium nitroprusside (SNP) and 1H (1,2,3) oxadiazole (4, 3‐a) quinoxalin‐1‐one (ODQ) were purchased from Sigma‐Aldrich Chemical Co. (St Louis, MI). Western blotting primary antibodies against Giα‐2 (sc‐13534), Giα‐3 (sc‐262), eNOS (sc‐654), dynein IC1/2 (sc‐13524), p22^phox^ (sc‐11712), Nox‐1 (sc‐5821), secondary antibodies goat anti‐mouse IgG horseradish‐peroxidase (HRP) conjugate (sc‐2005), Donkey anti‐goat IgG horseradish‐peroxidase (HRP) conjugate (sc‐2020), and enhanced chemiluminescence (ECL) detection system kits were purchased from Santa‐Cruz Biotechnologies (Santa Cruz, CA). Anti‐p47‐phox (07‐497), and Nox‐4 (ABC271) primary antibodies were purchased from EMD Millipore. Anti‐Nox‐2/gp91phox (ab129068) primary antibody was purchased from Abcam Inc. (ON, Canada). Secondary antibody Goat Anti‐Rabbit IgG (H+L)‐HRP conjugate was purchased from BIO‐RAD. The L‐(4,5‐^3^H) thymidine was from PerkinElmer Inc. (Waltham, MA). All other chemicals used in the experiments were purchased from Sigma‐Aldrich.

### Animal treatment

Male SHR (8‐week‐old) and age‐matched WKY rats were purchased from Charles River Laboratories International Inc. (St‐Constant, Quebec, Canada). Animals were maintained at room temperature with free access to water and regular rat chow in 12‐h light/dark cycles. Rats were left for 2 days for acclimatization. SHR and age‐matched WKY rats were injected intraperitoneally with SNP (0.5 mg/kg body weight) twice per week for 2 weeks in 0.01 mol/L sodium phosphate buffer, pH 7.0, containing 0.05 mol/L NaCl. The control WKY rats and SHR received vehicle. The blood pressure (BP) was monitored twice a week by using the CODA noninvasive tail‐cuff method, according to the recommendation of American Heart Association (Kurtz et al. 2005). At the end of the 10th week, after taking the blood pressure, the rats were euthanized by decapitation after CO_2_ exposure. Thoracic aortas and hearts were removed. Some aortas were used for cell culture. All the animal procedures used in the present study were approved by the Comité de Déontologie de l'Expérimentation sur les Animaux (CDEA) of the University of Montreal (Approval no. 99050). The investigation conforms to the “Guide for the Care and Use of Laboratory Animals” published by the US National Institutes of Health (NIH) (Guide, NRC 2011).

### Cell culture and incubation

VSMC from control and SNP‐treated SHR and their age‐matched WKY rats were cultured from aortas as described previously (Anand‐Srivastava et al. 1982). The purity of the cells was checked by immunofluorescence technique using α‐actin as described previously (Liau and Chan 1989). These cells were found to contain high levels of smooth muscle‐specific actin. The cells were plated in 75 cm^2^ flasks and incubated at 37°C in 95% air and 5% CO_2_ humidified atmosphere in Dulbecco's modified Eagle's medium (DMEM) (with glucose, l‐glutamine and sodium bicarbonate) containing antibiotics and 10% heat‐inactivated fetal bovine serum (FBS). The cells were passaged upon reaching confluence with 0.5% trypsin containing 0.2% EDTA and utilized between passages 3 and 10. Confluent cells were then starved by incubation for 12 h in DMEM without FBS at 37°C to reduce the interference by growth factors present in the serum. To examine the effect of ODQ on SNP‐induced decreased expression of Giα proteins, the cells were preincubated in the absence (control) or presence of ODQ (20 *μ*mol/L) for 24 h. After incubation, the cells were washed three times with PBS and lysed in 100 *μ*L of buffer (25 mmol/L Tris‐HCl, pH 7.5, 25 mmol/L NaCl, 1 mmol/L Na orthovanadate, 10 mmol/L Na fluoride, 10 mmol/L Na pyrophosphate, 2 mmol/L ethylene, bis(oxyethylenenitrolo)tetracetic acid 2 mmol/L ethylenediamine tetracetic acid, 1 mmol/L phenylmethylsulfonyl fluoride, 10 *μ*g/mL aprotinin, 1% Triton X‐100, 0.1% sodium dodecyl sulfate (SDS), and 0.5 *μ*g/mL leupeptin) on ice. The cell lysates were centrifuged at 12,000*g* for 5 min at 4°C. Protein concentration was measured with the Bradford assay (Bradford 1976). Cell viability was checked by the trypan blue exclusion technique and indicated that >90–95% cells were viable.

### Western blotting

The levels of protein expression and phosphorylation were determined by Western blotting as described previously (Hashim et al. 2006). After SDS‐PAGE, the separated proteins were transferred to a nitrocellulose membrane with a semidry transblot apparatus (Bio‐Rad Laboratories, Mississauga, Ontario, Canada) at 15 V for 45 min or a liquid transfer apparatus (Bio‐Rad Laboratories) at 100 V for 1 h. Membranes were blocked for 1 h at room temperature with 5% dry milk and incubated overnight with specific primary antibodies against different proteins in phosphate buffer solution (PBS) containing 0.1% Tween‐20 (PBST) overnight at 4°C. Dynein was used as loading controls in all experiment. The antibody‐antigen complexes were detected by incubating the membranes with horseradish‐peroxidase‐conjugated secondary antibodies for 1 h at room temperature. The blots were then washed three times with PBST before reaction with enhanced chemiluminescence (ECL). Quantitative analysis of the proteins was performed by densitometric scanning of the autoradiographs using the enhanced laser densitometer LKB Ultroscan XL and quantified using the gel‐scan XL evaluation software (version 2.1) from Pharmacia (Baie d′Urfé, Québec, Canada).

### Determination of superoxide anion (O_2_^−^) production and NADPH oxidase activity

Basal O_2_^−^ production and NADPH oxidase activity in VSMC were measured using the lucigenin‐enhanced chemiluminescence method with low concentration (5 *μ*mol/L) of lucigenin as described previously (Lappas et al. 2005; Saha et al. 2008). VSMC from control and SNP‐treated SHR and WKY rats were washed in oxygenated Kreb‐Hepes buffer, scraped and placed in scintillation vials containing lucigenin solution, and the emitted luminescence was measured with a liquid scintillation counter (Wallac 1409; PerkinElmer Life Science, St Laurent, Quebec, Canada) for 5 min. The average luminescence value was estimated, the background value subtracted and the result was divided by the total weight of tissue/proteins in each sample. The NADPH oxidase activity in the samples was assessed by adding 10^−4^ mol/L NADH (Sigma Chemical Co.) in the vials before counting. Basal superoxide‐induced luminescence was then subtracted from the luminescence value induced by NADPH.

### Determination of intracellular levels of hydrogen peroxide (H_2_O_2_), nitric oxide (NO), and peroxynitrite (ONOO^−^)

The levels of intracellular H_2_O_2,_ NO and ONOO^−^ produced in VSMC were measured using intracellular fluorescent probes, dichloro‐dihydro‐fluorescein diacetate (DCFH‐DA), diaminofluorescein‐2 diacetate (DAF‐2DA), and dihydrorhodamine 123 (DHR 123), respectively as described earlier (Iuchi et al. 2003; Li et al. 2014; Sarkar et al. 2017). Confluent VSMC, after washing twice with PBS, were incubated at 37°C for 1 h with 10 mmol/L DCFH‐DA for detecting H_2_O_2_, with both 10 mmol/L ol/L DAF‐2DA and 10^−6^ mol/L acetylcholine for detecting NO, and with 5 × 10^−3^ mol/L DHR 123 for detecting ONOO^−^, respectively. Cells were washed twice with PBS, and fluorescence intensities were measured by a spectrophotometer (TECAN infinite 200 PRO) with excitation and emission wavelengths at 495 nm and 515 nm for DCFH‐DA, 495 nm and 515 nm for DAF‐2DA, and 480 nm and 530 nm for DHR 123, respectively. Changes in fluorescence intensities were expressed as percentages of the values obtained in the WKY group (taken as 100%).

### Determination of cyclic guanosine monophosphate (cGMP) levels

cGMP levels were quantitatively measured by using an ELISA (enzyme‐linked immunosorbent assay) kit (Abcam, 330 Cambridge Science Park, Cambridge, CB40FL, UK) according to the manufacturer's instructions as described previously (Kang et al. 2013; Sarkar et al. 2017) and the concentration was expressed as picomoles per *μ*g of protein. All the ELISA experiments were repeated at least four times.

### Statistical analysis

Results are the mean ± SEM. Comparisons between groups were made with a one‐way analysis of variance (ANOVA) followed by the Tukey's Multiple Comparison Test, using GraphPad Prism 5 software. Results were considered statistically significant at values of *P *<* *0.05.

## Results

### SNP attenuates the development of high blood pressure in SHR

**Figure 1:**
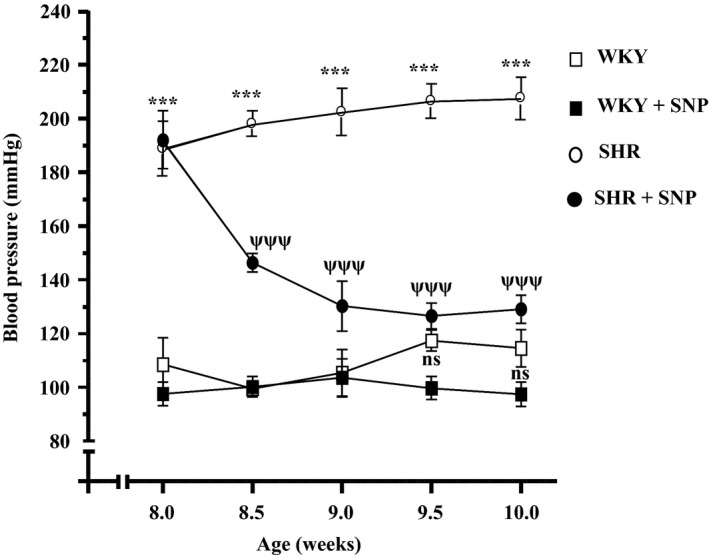
Effect of sodium nitroprusside (SNP) treatment on the development of high blood pressure (BP). Spontaneously hypertensive rats (SHR) and Wistar‐Kyoto (WKY) rats of 8 weeks old were injected intraperitoneally with SNP (0.5 mg/kg body weight) or vehicle twice per week for 2 weeks and BP was monitored twice weekly as described in the Materials and methods section. Values are mean ± SEM of six rats in each group. ***P <* *0.001 versus WKY, ^ψψψ^ *P *< 0.001 versus SHR, n.s. (not significant).

Since NO was shown to attenuate the enhanced expression of Giα proteins in VSMC from SHR (Sarkar et al. 2017) that is implicated in the pathogenesis of hypertension (Marcil et al. 1997), it was desirable to examine if treatment of SHR with SNP would attenuate the high BP. Results on BP profile are shown in Figure 1. Mean BP was significantly higher in SHR as compared to WKY rats (190 ± 5 vs. 110 ± 3 mmHg, *P *<* *0.001) and intraperitoneal injection of SNP to 8‐week‐old SHR (0.5 mg/kg body weight) twice per week decreased the BP in a time‐dependent manner and at 10 weeks, the BP was decreased by about 80 mmHg (130 ± 5 vs. 210 ± 5 mmHg, *P *<* *0.001). On the other hand, SNP treatment did not significantly decrease the BP in WKY rats (114 ± 7 vs. 97 ± 4 mmHg, *P *<* *0.063).

Furthermore, SNP treatment did not appear to have adverse effects on the health of animals in the study, because all rats treated with SNP maintained or gained weight during the period of the study (body weights at 10 weeks were as follows: WKY rats, 229 ± 15.2 g; SNP‐treated WKY rats, 234 ± 9.3 g; SHR, 228 ± 4.9 g; and SNP‐treated SHR, 223 ± 5.8 g).

### SNP treatment attenuates the enhanced expression of Giα proteins in VSMC from SHR

**Figure 2:**
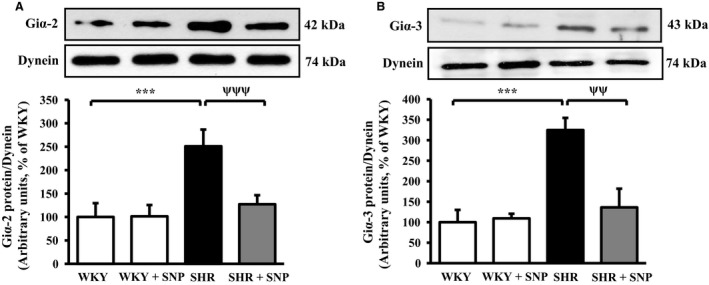
Effect of sodium nitroprusside (SNP) treatment on the expression of Giα proteins in aortic vascular smooth muscle cells (VSMC) from spontaneously hypertensive rats (SHR) and age‐matched Wistar‐Kyoto (WKY) rats. VSMC lysates from 10‐week‐old SHR and WKY rats with or without SNP treatment were subjected to Western blot analysis using specific antibodies against Giα‐2 and Giα‐3 (A and B, upper panels) as described in the Materials and methods section. Dynein was used as the loading control. The proteins were quantified by densitometric scanning (A and B, lower panels). Values are mean ± SEM of four independent experiments. The results are expressed as percentage of WKY (control) which has been taken as 100%. ***P <* *0.001 versus WKY; ^ψψ^ *P *< 0.01, ^ψψψ^ *P *< 0.001 versus SHR.

The role of enhanced expression of Giα proteins in the pathogenesis of hypertension has been shown (Anand‐Srivastava et al. 1991; Li and Anand‐Srivastava 2002; Marcil and Anand‐Srivastava 2001; Marcil et al. 1997). We earlier showed that C‐ANP_4‐23_ that interacts specifically with natriuretic peptide receptor‐C (NPR‐C) attenuated the development of high blood pressure in SHR through the inhibition of enhanced expression of Giα proteins (Li et al. 2014). To examine whether SNP‐induced attenuation of high BP in SHR is also attributed to its ability to decrease the enhanced expression of Giα proteins, the levels of Giα‐2 and Giα‐3 proteins were determined in aortic VSMC isolated from control and SNP‐treated SHR and WKY rats by Western blotting using specific antibodies against Giα‐2 and Giα‐3. The expression of Giα‐2 and Giα‐3 proteins was significantly augmented by about 150% and 220%, respectively in aortic VSMC (Fig. 2A and B) from SHR as compared with WKY rats and SNP treatment significantly attenuated the enhanced expression by about 85%. On the other hand, SNP treatment did not attenuate the expression of Giα‐2 and Giα‐3 proteins in VSMC from WKY rats.

### SNP decreased the expression of Giα proteins by cGMP‐independent mechanism in VSMC from SHR

**Figure 3:**
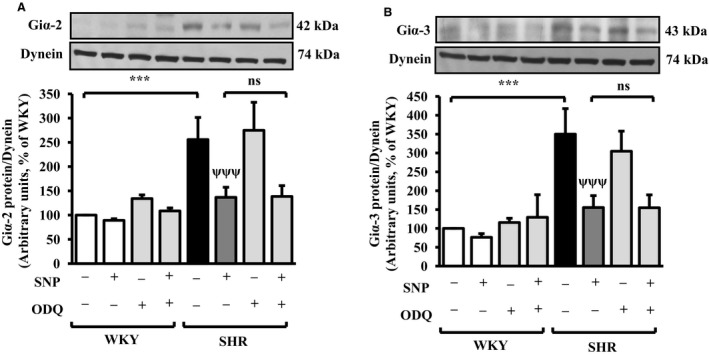
Effect of ODQ on sodium nitroprusside (SNP)‐induced decreased expression of Giα proteins in aortic vascular smooth muscle cells (VSMC) from spontaneously hypertensive rats (SHR) and age‐matched Wistar‐Kyoto (WKY) rats. Confluent VSMC from 10‐week‐old SHR and WKY rats treated with and without SNP were incubated in the absence or presence of ODQ (20 *μ*mol/L) for 24 h and cell lysates were subjected to Western blot analysis using specific antibodies against Giα‐2 and Giα‐3 (A and B, upper panels) as described in the Materials and methods section. Dynein was used as the loading control. The proteins were quantified by densitometric scanning (A and B, lower panels). Values are mean ± SEM of four independent experiments. The results are expressed as percentage of WKY (control) which has been taken as 100%. n.s. (not significant), ***P <* *0.001 versus WKY; n.s. (not significant), ^ψψψ^ *P *< 0.001 versus SHR

Since soluble guanylyl cyclase and cGMP pathways are implicated in most of NO‐mediated effects (Lane and Gross 1999; Ziolo et al. 2008), we investigated the role of cGMP in SNP‐induced attenuation of overexpression of Giα proteins in VSMC from SHR. To test this, the effect of ODQ, a specific inhibitor of soluble guanylyl cyclase (sGC), was examined on the expression of Giα proteins in VSMC from SHR and WKY rats treated with or without SNP. Results shown in Figure 3, indicate that SNP‐induced decreased expression of Giα‐2 (Fig. 3A) and Giα‐3 (Fig. 3B) in VSMC from SHR and WKY rats was not affected by pretreatment of cells with ODQ (20 *μ*mol/L). These results suggest that SNP‐induced decreased expression of Giα proteins involves cGMP‐independent mechanism.

### Effect of SNP on enhanced oxidative stress in aorta and VSMC from SHR

**Figure 4:**
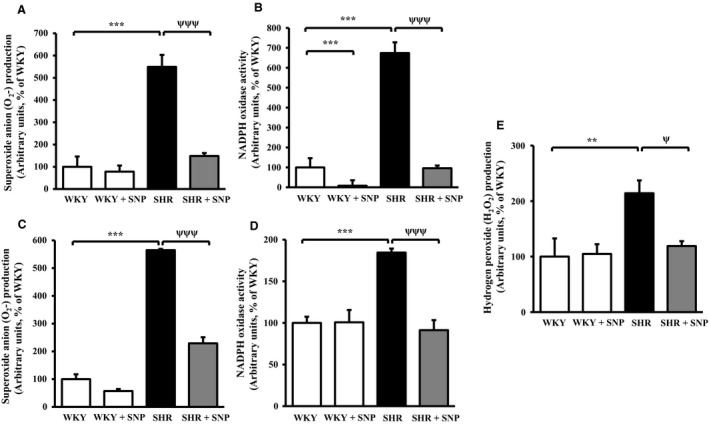
Effect of sodium nitroprusside (SNP) treatment on the production of superoxide anion (O_2_^−^), and NADPH oxidase activity and hydrogen peroxide (H_2_O_2_) levels in aorta, and aortic vascular smooth muscle cells (VSMC) from spontaneously hypertensive rats (SHR) and age‐matched Wistar‐Kyoto (WKY) rats. The O_2_^−^ production and NADPH oxidase activity were determined in VSMC (A and B), and aorta (C and D) and H_2_O_2_ levels (E) in VSMC from 10‐week‐old SHR and age‐matched WKY rats with or without SNP treatment as described in the Materials and methods section. Values are mean ± SEM of six independent experiments. The results are expressed as percentage of WKY (control) which has been taken as 100%. **P <* *0.01, ***P <* *0.001 versus WKY; ^ψ^ *P *< 0.05, ^ψψψ^ *P *< 0.001 versus SHR.

We earlier showed the implication of enhanced oxidative stress in overexpression of Giα proteins in VSMC from SHR (Gomez Sandoval and Anand‐Srivastava 2011; Lappas et al. 2005). To investigate if SNP treatment could also attenuate the augmented oxidative stress, which results in the attenuation of overexpression of Giα proteins, the levels of O_2_^−^ and NADPH oxidase activity were examined in aorta and aortic VSMC from control and SNP‐treated SHR and WKY rats. The results shown in Figure 4 indicate that the levels of O_2_^−^ were significantly higher by about 450% in aortic VSMC (Fig. 4A) and 465% in aorta (Fig. 4C) from SHR as compared with WKY and these augmented levels were attenuated by about 90%, and 70%, respectively by SNP treatment. Furthermore, the activity of NADPH oxidase that was enhanced by about 580% in aortic VSMC (Fig. 4B) and about 85% in aorta (Fig. 4D) from SHR as compared with WKY rats was also completely restored to WKY levels by SNP treatment. In addition, SNP also significantly decreased the NADPH oxidase activity in VSMC from WKY rats by about 90% (Fig. 4B).

We also examined the effect of SNP treatment on the levels of H_2_O_2_ that have been reported to augment the expression of Giα proteins in VSMC (Mbong and Anand‐Srivastava 2012). Results shown in Figure 4E demonstrate that the levels of H_2_O_2_ were also enhanced in VSMC from SHR by about 110% compared to WKY rats which were reduced to WKY levels by SNP treatment; however, the levels of H_2_O_2_ were not affected by SNP treatment in WKY rats.

### SNP treatment attenuates the expression of NADPH oxidase subunits in VSMC from SHR

**Figure 5:**
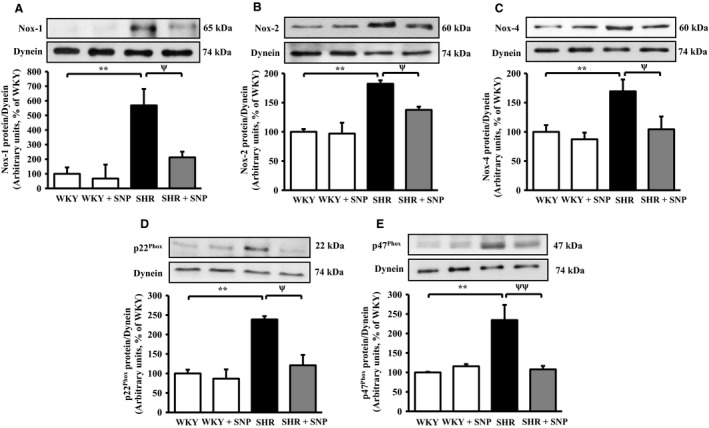
Effect of sodium nitroprusside (SNP) treatment on the expression of NADPH oxidase subunits in aortic vascular smooth muscle cells (VSMC) from spontaneously hypertensive rats (SHR) and age‐matched Wistar‐Kyoto (WKY) rats. VSMC lysates from 10‐week‐old SHR and WKY rats with or without SNP treatment were subjected to Western blot analysis using specific antibodies against Nox‐1 (A, upper panel), Nox‐2/gp91^phox^ (B, upper panel), Nox‐4 (C, upper panel), p22^phox^ (D, upper panel), and p47^phox^ (E, upper panel) as described in the Materials and methods section. Dynein was used as the loading control. The proteins were quantified by densitometric scanning (A–E, lower panels). Values are mean ± SEM of four independent experiments. The results are expressed as percentage of WKY (control) which has been taken as 100%. **P <* *0.01 versus WKY; ^ψ^ *P *< 0.05, ^ψψ^ *P *< 0.01 versus SHR.

To further examine if SNP‐induced decreased activity of NADPH oxidase in SHR is attributed to its ability to decrease the enhanced expression of different subunits of NADPH oxidase, the effect of SNP treatment on the expression of Nox‐1, Nox‐2/gp91^phox^, Nox‐4, p22^phox^, and p47^phox^ proteins was examined in VSMC from control and SNP‐treated SHR and WKY rats. The results shown in Figure 5 demonstrate that the levels of Nox‐1 (Fig. 5A), Nox‐2/gp91^phox^ (Fig. 5B), Nox‐4 (Fig. 5C), p22^phox^ (Fig. 5D), and p47^phox^ (Fig. 5E) were significantly augmented in VSMC from SHR by about 460%, 80%, 70%, 135%, and 130%, respectively, as compared to WKY rats, and this increase was restored to control WKY level by SNP treatment. However, SNP treatment was ineffective in altering the levels of these subunits in WKY rats.

### Effect of SNP treatment on the intracellular levels of NO, eNOS, cGMP, and ONOO^−^ in VSMC from SHR

**Figure 6:**
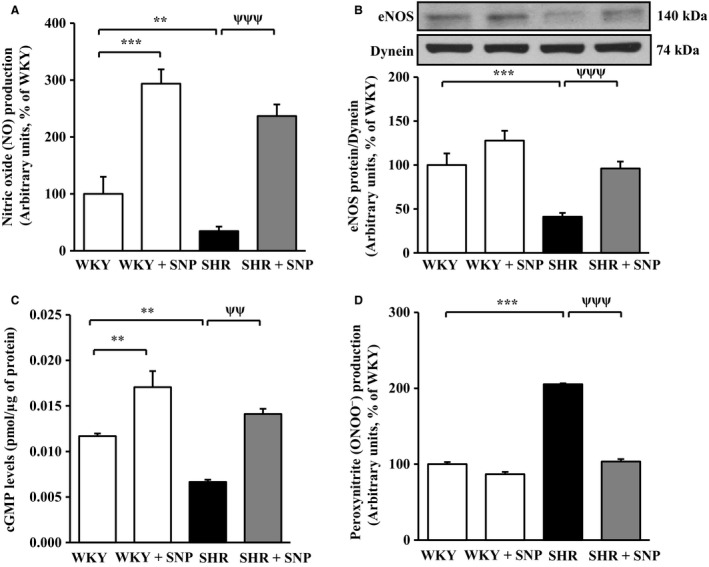
Effect of sodium nitroprusside (SNP) treatment on the intracellular levels of nitric oxide (NO), endothelial nitric oxide synthase (eNOS), cyclic guanosine monophosphate (cGMP), and peroxinitrite (ONOO^−^) in aortic vascular smooth muscle cells (VSMC) from spontaneously hypertensive rats (SHR) and age‐matched Wistar‐Kyoto (WKY) rats. Confluent VSMC from 10‐week‐old SHR and WKY rats with or without SNP treatment were incubated at 37°C for 1 h with fluorescent probes diaminofluorescein‐2 diacetate (DAF‐2DA) and dihydrorhodamine 123 (DHR 123) for the measurement of intracellular NO (A) and ONOO^−^ (D) levels, respectively and the levels cGMP (C) were determined by ELISA as described in the Materials and methods section. VSMC lysates from SHR and WKY rats with or without SNP treatment were subjected to Western blot analysis using specific antibody against eNOS (B, upper panels) as described in the Materials and methods section. Dynein was used as the loading control. The proteins were quantified by densitometric scanning (B, lower panels). Values are mean ± SEM of six independent experiments. The results are expressed as percentage of WKY (control) which has been taken as 100%. **P <* *0.01, ***P <* *0.001 versus WKY; ^ψψ^ *P *< 0.01, ^ψψψ^ *P *< 0.001 versus SHR.

Earlier studies have shown that VSMC from SHR exhibit decreased levels of intracellular NO, eNOS, and cGMP which may contribute to the development of hypertension (Fukuda et al. 1991; Li et al. 2014; Sarkar et al. 2017). To investigate if SNP‐induced attenuation of high BP in SHR is also due to the augmentation of the intracellular levels of NO, eNOS, and cGMP, we determined the levels of NO, eNOS, and cGMP in VSMC from control and SNP‐treated SHR and WKY rats. Results shown in Figure 6 indicate that the intracellular levels of NO (Fig. 6A), eNOS (Fig. 6B), and cGMP (Fig. 6C) were significantly attenuated by about 65%, 60%, and 40%, respectively in VSMC from SHR as compared to WKY rats and SNP treatment restored these decreased levels toward control levels. In addition, the intracellular levels of NO and cGMP were also increased in WKY rats by about 130% and 50%, respectively.

We earlier showed that VSMC from SHR exhibits increased levels of ONOO^−^ resulting in nitrosative stress (Li et al. 2014). To investigate if SNP treatment could also attenuate the increased levels of ONOO^−^ in VSMC from SHR, the effect of SNP treatment on the levels of ONOO^−^ was determined in VSMC from control SHR and WKY rats and the results are shown in Figure 6D. As reported earlier (Li et al. 2014), the levels of ONOO^−^ were significantly increased by about 100% in VSMC from SHR compared with WKY rats which were attenuated to control levels by SNP treatment. However, the levels of ONOO^−^were not significantly attenuated by SNP treatment in VSMC from WKY rats.

## Discussion

Earlier studies have shown that the supplementation of exogenous nitrite, an inert metabolic by‐product of the oxidation of NO (Bryan 2009), attenuates blood pressure (Beier et al. 1995; Classen et al. 1990) and endothelium‐dependent relaxation in isolated aortae of SHR by activating the eNOS‐NO‐soluble guanylyl cyclase (sGC)‐cGMP pathway (Ling et al. 2015). In addition, there has been one study showing that SNP, a potent NO donor attenuates high BP in SHR (Akiba et al. 1995), However, in the present study, we report for the first time that SNP‐induced attenuation of enhanced expression of Giα proteins and nitroxidative stress contributes to the reduction of high BP in SHR.

The treatment of 8‐week‐old SHR with SNP intraperitonealy (twice per week) decreased the BP in a time‐dependent manner and at 10 weeks, the BP was decreased by about 80 mmHg. However, this treatment did not significantly reduce the BP in WKY rats suggesting that SHR may be simply more sensitive to SNP than WKY rats. In addition, the overexpression of Giα‐2 and Giα‐3 proteins in VSMC from SHR was also attenuated by SNP treatment whereas the basal expression of these proteins in WKY rats was not affected. These results suggest that SNP‐induced attenuation of overexpression of Giα proteins may be responsible for the reduction of high BP in SHR. In this regard, the inactivation of Giα proteins by intraperitoneal injection of pertussis toxin into prehypertensive SHR has also been shown to attenuate the development of high BP in SHR (Li and Anand‐Srivastava 2002). In addition, C‐ANP_4‐23_‐induced attenuation of the development of high BP in SHR was also shown to be associated with the inhibition of overexpression of Giα proteins (Li et al. 2014).

We earlier showed that the attenuation of overexpression of Giα proteins in VSMC from SHR by NO donors was through a cGMP‐independent mechanism (Sarkar et al. 2017). In the present study also, we demonstrate that the attenuation of augmented expression of Giα proteins by in vivo treatment of SHR with SNP involves cGMP‐independent mechanism because ODQ, an inhibitor of soluble guanylyl cyclase (sGC), was unable to reverse the attenuated expression of Giα proteins induced by SNP.

We also demonstrate that VSMC from SHR exhibit enhanced levels of O_2_^−^, NADPH oxidase activity and augmented expression of NADPH oxidase subunits. These results are in accordance with our earlier studies and the studies of other investigators (Lappas et al. 2005; Lodi et al. 2006; Zalba et al. 2000). Furthermore, the fact that in vivo treatment of SHR with SNP attenuated the enhanced levels of O_2_^−^, H_2_O_2_, NADPH oxidase activity, and NADPH oxidase subunits suggests that SNP‐evoked reduction of high BP may be due to the inhibition of oxidative stress. Our results are in accordance with the study of Prabha et al. (1990) who have also reported that patients with uncontrolled hypertension showed a higher level of H_2_O_2_ and O_2_^−^, and these high levels had reverted to normal levels after the control of high BP. Furthermore, the contribution of enhanced oxidative stress in the overexpression of Giα proteins in VSMC from SHR has been shown (Ali El‐Basyuni et al. 2016; Li et al. 2010). In addition, our recent study also demonstrated that NO donors; SNAP and SNP inhibited the overexpression of Giα proteins in VSMC from SHR through inhibiting reactive oxygen species (ROS) and ROS‐mediated signaling (Sarkar et al. 2017). Taken together, it may be suggested that SNP through the inhibition of oxidative stress attenuates the enhanced expression of Giα proteins and thereby results in the attenuation of high BP.

A decreased production/bioavailability of NO has been shown to be associated with hypertension (Chou et al. 1998; Crabos et al. 1997; Dubois 1996). In addition, the fact that eNOS knockout mice exhibit increased BP (Van Vliet and Chafe 2007) and the inhibition of NO synthase by L‐NAME augmented BP (Di Fusco and Anand‐Srivastava 2000) further supports the implication of decreased levels of NO in the elevated BP. Our results showing that the levels of NO and eNOS were decreased in VSMC from SHR are in accordance with our earlier study and other studies (Elks et al. 2011; Li et al. 2014; Sarkar et al. 2017; Wiemer et al. 2001; Zhang et al. 2013) and may be attributed to the overproduction of O_2_^−^ (Li et al. 2014). Furthermore, the fact that SNP treatment augmented the reduced levels of eNOS and NO in VSMC from SHR suggests that SNP‐induced reduction in high BP may also be due to its ability to enhance the levels of NO. In this regard, the supplementation of exogenous nitrite derivatives has been reported to exert antihypertensive effects in SHR, as well as in animals with two‐kidney, one‐clip (2K‐1C), or salt‐induced hypertension models due to the elevation of NO levels (Beier et al. 1995; Carlstrom et al. 2011; Ling et al. 2015; Montenegro et al. 2011). Furthermore, chronic treatment of SHR with sodium nitrite was shown to improve endothelium‐dependent relaxation in addition to its antihypertensive effect through the enhanced activation of eNOS (Ling et al. 2016). However, whether in vivo treatment of SHR with SNP also improves endothelium‐dependent relaxation remains to be investigated.

In addition, our results showing that VSMC from SHR exhibit augmented levels of ONOO^−^ are also in accordance with our earlier study and the studies of other investigators who also showed augmented levels of ONOO^−^ in aorta, mesenteric arteries, and kidney from SHR (Elks et al. 2011; Li et al. 2014; Mason et al. 2006; Zhang et al. 2013). However, we report for the first time that in vivo treatment of SHR with SNP decreases the augmented levels of ONOO^−^ in VSMC and suggests that SNP‐induced attenuation of high BP in SHR may also be attributed to its ability to inhibit the nitrosative stress. In support of this notion, Mason et al. (2015) also showed the implication of enhanced levels of NO and decreased levels of ONOO^−^ in atorvastatin‐induced reduction of high BP in SHR with diabetes.

In this study, we also show that the intracellular levels of cGMP were significantly lower in VSMC from SHR as compared to VSMC from WKY rats which may be due to the decreased levels of NO because in vivo treatment of SHR with SNP that elevates the intracellular levels of NO also augmented the levels of cGMP. These results are consistent with our previous report showing that NO donor, SNAP, augmented the intracellular levels of cGMP in VSMC from SHR and WKY rats (Sarkar et al. 2017). Fukuda et al. (1991) also showed that the levels of cGMP were lower in mesenteric arteries from 8‐, 12‐, and 20‐week old SHR compared to WKY rats and are associated with the development of hypertension in SHR (Chiu et al. 1991). Furthermore, since both SNP as well as cGMP have been reported to decrease high blood pressure in SHR (Chiu et al. 1991; Fukuda et al. 1991), it may be possible that SNP‐induced attenuation of high blood pressure in SHR is attributed to its ability to augment the intracellular levels of cGMP. The fact that SNP treatment attenuated the augmented levels of O_2_^−^ and ONOO^−^ and increased the intracellular levels of NO and cGMP in VSMC from SHR further suggests that SNP‐induced attenuation of high BP may be attributed to the increased levels of cGMP and decreased nitroxidative stress. In this regard, the inhibition of nitrosative stress was also shown to contribute to C‐ANP_4‐23_‐induced attenuation of high BP in SHR (Li et al. 2014).

In conclusion, the present study demonstrates for the first time that SNP attenuates high BP in SHR through the augmentation of cGMP levels and attenuation of overexpression of Giα proteins and nitroxidative stress. It may also be suggested that NO‐evoked decreased expression of Giα proteins may be an important additional mechanism through which SNP regulates BP.

## Conflict of Interest

None declared.
